# Vaping induces a marked pro-inflammatory state within the lung, which is at least partially due to its immunomodulatory effects

**DOI:** 10.3389/fimmu.2026.1759361

**Published:** 2026-03-10

**Authors:** Jeffrey G. Shipman, Kimberly Mundy, Rob U. Onyenwoke, Vijay Sivaraman

**Affiliations:** 1 Department of Biological & Biomedical Sciences, North Carolina Central University, Durham, NC, United States; 2 Biomanufacturing Research Institute and Technology Enterprise (BRITE), North Carolina Central University, Durham, NC, United States; 3 The Julius L. Chambers Biomedical/Biotechnology Research Institute, North Carolina Central University, Durham, NC, United States

**Keywords:** immune suppression, lung, MDSC (myeloid-derived suppressor cell), neutrophil, vaping

## Abstract

**Introduction:**

E-cigarette use or “vaping” exposes the lungs to a large array of chemicals, flavors and nicotine. Initially marketed as a healthier alternative to tobacco use, research has since begun to demonstrate vaping plays a role in immune dysfunction and inflammation, with both similarities and differences in effects observed compared with tobacco use. One potential mechanism of immune dysfunction is related to the regulation of monocyte-derived suppressor cells (MDSCs). MDSCs are a heterogenous population of cells that are generated and expand during inflammation and infection and are known and are commonly described as having immunosuppressive roles. MDSCs play an important role in modulating and suppressing immune response. However, vaping exposure may, in part, be associated with modifications in MDSC populations and inflammatory processes. Thus, we questioned whether vaping modulates MDSCs, potentially resulting in alterations in inflammatory cells and mediators in vivo.

**Methods:**

Briefly, male and female mice were vaped with JUUL Menthol (3% nicotine) pods using the SCIREQ InExpose system for 4 weeks. After 4 weeks, the mice were then exposed to lipopolysaccharide (LPS) to mimic a bacterial infection. Post-infection (24 hours) the mice were sacrificed, and bronchoalveolar lavage fluid (BALF) was collected.

**Results:**

Vaped mice that were challenged with LPS demonstrated a decrease in macrophage numbers and significantly lower levels of granulocytic/ polymorphonuclear myeloid-derived (PMN) -MDSC compared to mice treated with LPS alone or only vaped. In contrast, monocytic (M) -MDSC numbers increased in the vaped and LPS challenged mice compared to the mice only treated with LPS in females while in males exhibited a decrease in vape+LPS groups compared to LPS alone. When comparing these results to the inflammatory data, several cytokines and chemokines linked to M-MDSC development and function were expressed after only LPS treatment but not by the dual vape+LPS treatment. However, exceptions were IFN-γ and RANTES/CCL5, which were increased after the dual treatment.

**Discussion:**

These data suggest that the variation in cytokine and chemokine levels is associated with changes in M-MDSC populations.

## 1 Introduction

“Vaping” is defined as the use of the e-cigarette (E-cig) along with an e-liquid composed of propylene glycol and vegetable glycerin (at varying ratios) along with added flavor components both with and without nicotine. Recently emerged in the past 15 years, E-cig use now has been shown to have significant impact on many different populations. In particular, its most troublingly usage has been recorded among younger (adolescent) people and has been demonstrated to play a role in immune dysfunction and inflammation (1–3). Currently much is known regarding the traditional combustion of tobacco for the purpose of smoking, which serves as the major preventable risk factor for the development of lung disease and susceptibility to pulmonary disease due to microbial challenge. E-liquids were initially shown to carry limited chemicals (i.e., PG/VG vehicle, tobacco and flavorants). However, when vaping, the user similarly inhales a large array of novel chemicals due to physiochemical changes that occur during the vaporizing process (4). Vaping is also suggested to have negative health consequences, which can include immunomodulatory effects associated with the use of the e-liquid (5–9). One example of a negative consequence is that vaped alveolar macrophages (AM) demonstrate a phagocytosis defect, which equates to a key deficit of the lung innate immune response (10). This characteristic is key to the innate immune response in the lung. In addition, vaping also leads to a pro-inflammatory response from human neutrophils, with increases in neutrophil elastase and MMP-9 activity, and p38 MAPK activation (11). At the same time, E-cig use also appears to be linked with immunosuppression in the lower airways of vapers (12, 13). Inhaled e-liquid aerosols are believed to be deposited in these airway areas, that is, in the bronchoalveolar region (14). Thus, macrophages and neutrophils, which are two of the major inflammatory cell types involved in lung defense (15) and situated within this lower airway region, are likely affected as described immediately above (16). Indeed, evidence demonstrates that E-cigs exert some pro-inflammatory effects on human AMs (17), with the referenced study illustrating exposing AMs to a condensed e-liquid vapor increasing reactive oxygen species (ROS) production, again significantly inhibiting phagocytosis (an immunosuppressive effect) and increasing levels of cytokines such as IL-6 and TNF-α (pro-inflammatory cytokines) which are general markers of inflammation.

As such, we propose that vaping likely modulates the immune system, in part, by upregulation and activation of circulating myeloid-derived suppressor cells (MDSCs) (18, 19). MDSCs are a heterogeneous population of activated myeloid cells that possess a potent immunosuppressive ability (20). By definition, MDSCs are immature in nature, can be monocytic (M) -MDSC, then differentiating into macrophages or dendritic cells, or granulocytic/polymorphonuclear myeloid-derived (PMN) -MDSC, which are similar to neutrophils (21). Thus, MDSC dysregulation can cause significant collateral inflammatory damage to host tissues (22, 23). One mechanism of harm likely related to MDSC response due to vaping is the inhibition of the efferocytic (the phagocytosis and engulfment of dead cells) abilities of primary macrophages, which decreases bacterial clearance (24) and skews toward macrophage activation (25, 26). Previous studies have shown that vaping can influence immune regulatory pathways and inflammatory biomarkers, which provides context for the exploration of changes in MDSC populations within oral and lung tissues (27, 28). Thus, we decided to investigate whether vaping imparts a similar pro-inflammatory/immunomodulatory profile *in vivo*.

Calcium (Ca^2+^) signaling is specifically linked to MDSCs but typically studied within the context of cancer models. However, MDSCs have not been thoroughly investigated with regards to lung diseases. Under normal circumstances, MDSC generation and activation is mediated by CaMKK2 (Calcium/calmodulin-dependent protein kinase kinase 2). Ca^2+^ and Calmodulin bind to CaMKK2, resulting in activation that promotes MDSC generation and immunosuppressive function (29). Ca^2+^ is typically localized in the cytosol. Therefore, we wanted to focus on pathways that are linked to increased levels of intracellular Ca^2+^. Based on our previous research, we chose to focus on the release of intracellular Ca^2+^ stores as a means of increasing cytosolic Ca^2+^ levels. In particular, we sought to inhibit the release of Ca^2+^ from the endoplasmic reticulum using 2-aminoethoxydiphenyl borate (2-APB), a known Ca^2+^ inhibitor (30).

Herein we have built upon our previous *in vivo* data illustrating that vaping exacerbates lung cellularity and inflammation in the presence of an infection (an LPS challenge) (31) and demonstrate significant differences within pulmonary immune cell profiles among mice that: are vaped alone, receive an LPS challenge alone or that are both vaped and receive the LPS challenge. Further, we explore the apparent cytokine burden of these three (3) groups to expand upon this significant finding of drastically different cellularity among these three particular treatment groups. Finally, we compare these data between male and female mice to determine if there is a sex-dependent difference in response to these exposures. To our knowledge, these data are completely novel and speak to the paucity of data currently available on the pulmonary consequences of vaping and indicate that vaping presents a dual modality of potentially leading to the conundrum of both a pro-inflammatory response and immune suppression.

## 2 Materials and methods

### 2.1 Mice and treatments

Mice were obtained from Jackson Laboratories (Bar Harbor, ME, USA). Young adult C57-BL/6J mice (6- to 8-week-old male and female) were utilized for all experiments. After receiving, mice were allowed to acclimate and recover from shipping stress for at least 1 week in the NCCU Animal Resource Complex, which is accredited by the American Association for Accreditation of Laboratory Animal Care. All animal care and use were conducted in accordance with the Guide for the Care and Use of the Laboratory Animals (National Institutes of Health), and mice were maintained at 25 °C and 15% relative humidity with alternating 12 h light/dark periods.

**Figure 1:**
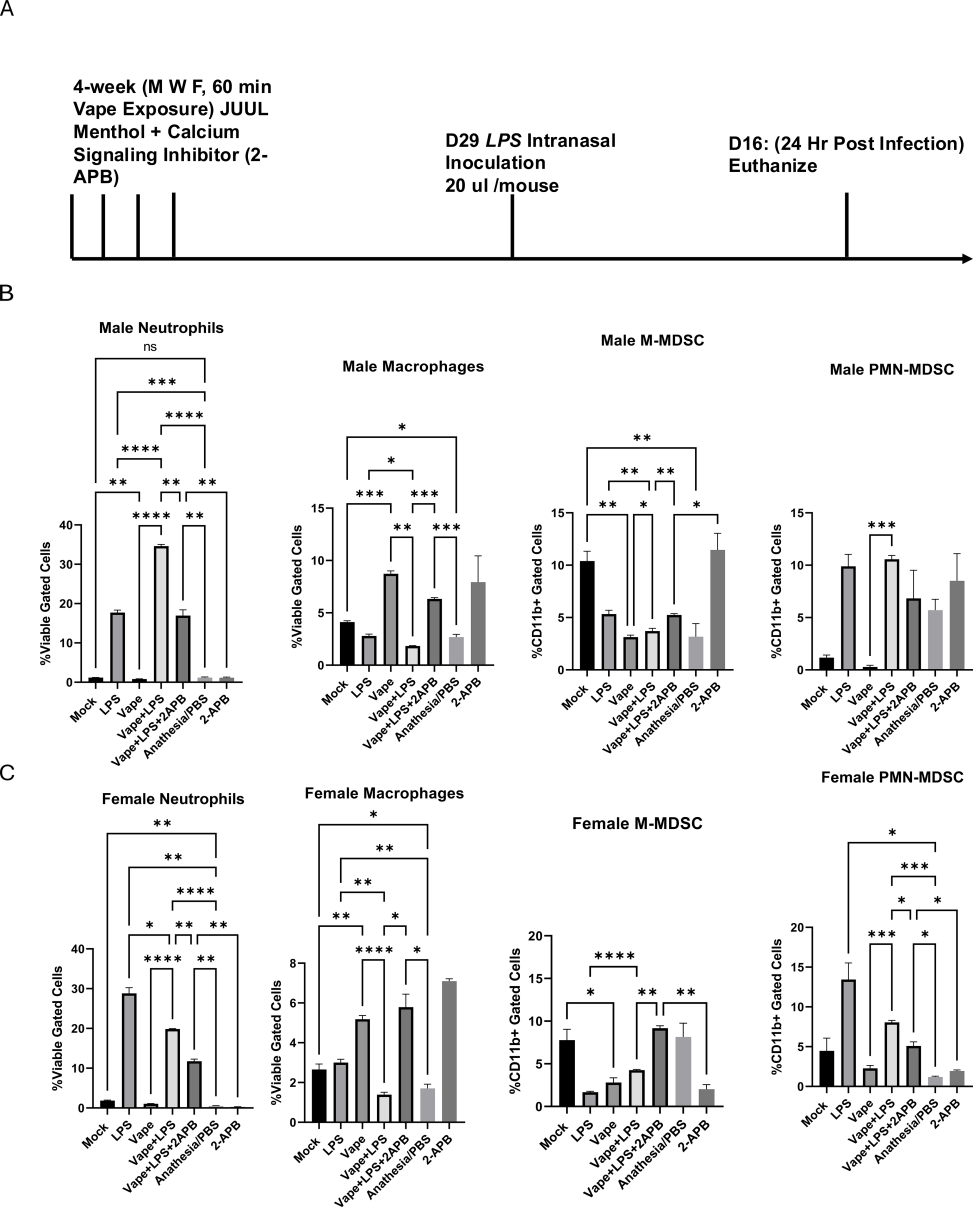
**(A)** An acute *in vivo* model of vaping, mice received vape exposures (JUUL “Menthol”) using a well-described puff topography of 2 puffs/min (8 s/110 mL/puff, flow rate of 2 L/min) for 1 hour per day, 3 days a week for 4 weeks, followed by an LPS (IN) challenge and were then sacrificed to obtain BALF. n = 8 mice per treatment group. **(B)** BALF was collected from male mice exposed to Mock, LPS, vape alone, vape+LPS, vape+LPS+2-APB, anesthesia/PBS, and 2-APB conditions. Stained cells were identified as macrophages, neutrophils, M-MDSCs and PMN-MDSCs. **(C)** BALF was collected from female mice exposed to Mock, LPS, vape alone, vape+LPS, vape+LPS+2-APB, anesthesia/PBS, and 2-APB conditions. Stained cells were identified for macrophages, neutrophils, M-MDSCs and PMN-MDSCs. Data are presented as mean ± SEM. Unpaired t-tests with Welch’s correction were used to determine statistical significance, which was indicated by the following p-values: P<0.05*, P<0.01 **, P<0.001***, P<0.0001****. ns, Not Significant.

Once acclimated, mice were vaped using a SCIREQ InExpose system via nose only method (SCIREQ Respiratory Equipment, Montreal, QC, Canada) and “Menthol” JUUL pods. Manufacturer’s label for JUUL pods indicates ingredients include: vegetable glycerin (VG), propylene glycol (PG), nicotine, flavoring and benzoic acid, with each pod containing 0.7 mL of the flavored fluid at 3% nicotine. We (32) and other groups (33, 34) have performed detailed chemical analyses (gas chromatography-mass spectrometry (GC-MS)) of multiple, different lots of JUUL pods and other e-liquid products. Results have been consistent and have been published. Using our previous acute *in vivo* model of vaping, mice received the vaping exposures using a well-described puff topography of 2 puffs/min (8 s/110 mL/puff, flow rate of 2 L/min) for 1 hour per day, 3 days a week for 4 weeks. Human vaping exposure varies per person, however what is typically seen in adolescents is a binge exposure throughout the week. Using this knowledge, we modeled a lesser binge exposure following day on, day off exposure. Four (4) weeks was chosen as the max amount of time considered for a subacute exposure in order to generate a more robust response than our previous data (31). During the 4 weeks of exposure, appropriate mice were given 10 µl of a 500 µM stock of 2-APB (a Ca^2+^ channel inhibitor) as outlined in our previous paper (31). This was chosen because increases in intracellular Ca^2+^ signaling has been seen in cells vaped with JUUL Menthol in our previous research. Following treatments, the appropriate group of mice (each consisting of 4 mice) was anesthetized via an i.p. injection of ketamine (100 mg/kg) and xylazine (50 mg/kg) using a 22-gauge syringe. The protocol for ketamine and xylazine anesthesia is approved in our protocol by the NCCU Animal Care and Use Committee and routinely used with no discernable cardiopulmonary distress, mortality or effects observed by HR, CO_2_ or O_2_ measurements. Data from this protocol have recently been published (31, 35, 36). The ketamine and xylazine anesthesia allows for a dropwise intranasal (IN) administration of 20 µl lipopolysaccharide (LPS; 0.4 mg/mL, *E. coli*, Serotype R515) to mimic a bacterial infection (Sigma-Aldrich; St. Louis, MO, USA; **Figure 1A**). LPS was chosen to mimic a bacterial infection due to it being present and secreted by Gram negative bacteria. Mice were then sacrificed after 24 h, and bronchoalveolar lavage fluid (BALF) was collected. Additionally, PBS was used as a negative control for comparison of mock vs. vaped. At time points of experimental completion, the mice were humanely euthanized using CO_2_ asphyxiation and cervical dislocation, as per our accepted animal protocol and Animal Resource Complex housing guidelines and conditions.

### 2.2 Cellular identification

Three color flow cytometry analysis was performed using a CytoFLEX Flow Cytometer (Beckman Coulter, FL, USA) with dual laser excitation (488 nm and 638 nm). Cells isolated from BAL were stained with fluorescent-tagged antibodies as per the manufacturers’ instructions. LY6C-AF488(Clone: HK1.4 Cat:53-5932-82), Ly6G-PE(Clone:1A8-Ly6g Cat:12-9668-82), F4/80-AF700(Clone: BM8 Cat:56-4801-82),CD11b-APC(Clone:M1/70 Cat:17-0112-82), and eBioscience Fixable Viability Dye eFluor 450(L23105) were purchased from Thermo Fisher Sci (eBiosciences). Antibodies were chosen to sort mouse macrophages and MDSC similar to previously published work (**Table 1**) (37). BALF was isolated from mice, Supernatant was removed and Red Blood Cell Lysis buffer was added to cells and incubated at room temperature for 5 min. with shaking. Following RBC lysis, cells are washed in using PBS and centrifuges at 400 g. PBS was discarded, cell pellet is resuspended in flow staining buffer (PBS containing 2% Fetal Bovine Serum) and incubated on ice for 10 min. Cells were washed using flow staining buffer, the pellet was resuspended in 50 µl of stain cocktail mix containing our selected antibodies and incubated at 4 °C in the dark for 30 min. Following incubation, cells were washed and resuspended in at 200-300 µl of flow staining buffer. Cells are read using CytoFLEX within 1 h. Representative flow gating strategy and figures are demonstrated in **Supplementary Figure 1A**.

**Table 1:** Antibody panel for flow cytometry.

Cell type	Cell surface markers
Neutrophils	F4/80^-^Ly6G^+^
Macrophages	F4/80^+^CD11b^mid^
M-MDSCs	CD11b^+^Ly6G^-^Ly6C^hi^
PMN-MDSCs	CD11b^+^Ly6G^+^Ly6C^lo^

### 2.3 Bicinchoninic acid protein assay

At experimental endpoints, mice were sacrificed, BALF was collected, supernatants were isolated and total protein was analyzed. Clarified BALF total protein was evaluated using Pierce™ BCA protein assay kit purchased from Thermo Fisher Scientific (Waltham, MA, USA).

### 2.4 Inflammatory markers

BALF was centrifuged (500 g at 4 °C for 10 min) to obtain a cell-free pellet. The cell-free BALF fraction was concentrated to ~10 times the original volume using Amicon Ultra 3 kDa centrifugal filters (Millipore Sigma; UFC200324) following the manufacturer’s instructions. A MILLIPLEX (#MCYTOMAG-70K-PMX, Millipore-Sigma) panel of 25 cytokines was then utilized along with the clarified BALF and a Luminex LX200 system (38). The lower detection limit for the previously mentioned kit is 1 picogram per milliliter (pg/mL) or picogram per microgram of total protein. **Table 2** outlines the groupings of inflammatory proteins analyzed. GraphPad Prism 9 (La Jolla, CA, US) was used to generate data from Luminex quantification.

**Table 2:** Classification of quantified inflammatory proteins.

Classification	Inflammatory proteins
Macrophage Cytokines	TNF-α, IL-6, IL-1α, IL-1β
MDSC Generation and Expression	IL-1β, IL-6, VEGF
MDSC Activation	TNF-α, IL-6, INF-γ, IL-1β
MDSC Migration	MCP-1, MIP-1α, MIP-1β, RANTES

### 2.5 Histopathology

At the time points of experimental completion, mice were sacrificed. Lungs were then inflated with 1 mL of 10% neutral buffered formalin, removed and suspended in 10% formalin for 12 h. Lungs were washed once in PBS and then immersed in 70% ethanol. Tissues were then embedded in paraffin, and three 5-μm sections (200 μm apart) per lung were stained with hematoxylin/eosin (H&E). Sections were evaluated blindly for gross pathology at 40x and 100x total magnification. Acute lung injury scoring was completed at 400x using the procedure outlined in the following paper (39). Briefly, at least 20 pictures were randomly taken of each lung at 400x total magnification, additionally pictures taken were evenly distributed among sections to avoid bias To generate the acute lung injury score, pictures were scored using 5 independent variables weighted according to relevance. These variables were as follows: (1) Neutrophils in the alveolar space, (2) Neutrophils in the interstitial space, (3) Hyaline membranes, (4) Proteinaceous debris filling the airspaces and (5) Alveolar septal thickening. These variables were then summed and normalized by the number of fields to create a lung injury score between zero and one.

### 2.6 Statistics

All statistical assessments were performed using GraphPad Prism 9 (La Jolla, CA, US). All experimental samples were run in at least triplicate. Statistical analyses were performed using unpaired t-tests with Welch’s correction for pairwise comparisons. The experimental design is multi-variable, so these analyses were conducted to explore and assess the differences between specific conditions. Significance thresholds for tests were reported using the following values: P<0.05*, P<0.01 **, P<0.001***, P<0.0001****.

## 3 Results

### 3.1 Vaping and LPS challenge alter cellularity and cell phenotype within the mouse lung

Our previous *in vivo* data illustrate that vaping exacerbates lung cellularity and inflammation in the presence of a mock infection, that is, when lipopolysaccharide (LPS) is delivered intranasally (compared to LPS alone) (31). In particular, our data indicate that vaping followed by an LPS challenge results in pulmonary neutrophilia (31). Based upon this previous study, we questioned how vaping might mechanistically alter inflammation characterized by cellularity of the lung. To this end, we evaluated the presence of inflammatory cells within the BALF of exposed mice using a flow cytometry antibody panel (**Table 1**) to distinguish viable F4/80^+^CD11b^+^ cells as well as macrophages (F4/80^+^CD11b^mid^), neutrophils (F4/80^-^Ly6G^+^) and MDSCs, which were evaluated as M-MDSC (CD11b^+^Ly6G^-^Ly6C^hi^) or PMN-MDSC (CD11b^+^Ly6G^+^Ly6C^lo^). Representative data of the flow cytometry analysis are provided (**Supplementary Figure 1**).

In this study, we were interested in whether vaping might affect the frequency of immune cell populations, particularly neutrophils and MDSCs, and how these cells types are associated with previously observed histological changes (31). Herein we exposed 6- to 8- week old male and female mice to vape (JUUL menthol) only, LPS (IN) only or vape followed by LPS. During our analysis, we observed that male mice vaped and then challenged with LPS carry significantly decreased levels of macrophages (p<0.01, p<0.05) as well as significantly increased levels of neutrophils (p<0.0001, p<0.0001) as compared to mice that are only vaped or only challenged with LPS (**Figure 1B**). At the same time, mice that were vaped and challenged with LPS had similar levels of PMN-MDSC compared to mice that only received an LPS challenge (**Figure 1B**). This result in PMN-MDSC was in contrast to M-MDSC cell numbers, where mice that were vaped and challenged with LPS had a slightly significant (p<0.05) lower levels of M-MDSC compared to mice that only received the LPS challenge. Interestingly enough in the male mice, the anesthesia/PBS and mock groups were significantly different in terms of the macrophage and M-MDSC cell numbers, suggesting that either the anesthesia or PBS vehicle/control may also play an unintended role in our results.

When looking at the females, mice vaped and then challenged with LPS demonstrated a decrease in neutrophils compared to LPS alone. Similar trends seen in neutrophils were observed in PMN-MDSC population. Lower levels of macrophages were present in mice vaped and challenged with LPS. (**Figure 1C**) These data suggest notable changes in cell populations within lungs that are exposed to vaping or LPS alone compared with lungs exposed to the dual treatment, vape+LPS challenge.

2-Aminoethoxydiphenyl borate (2-APB) is a known inhibitor of the Ca^2+^ signaling component IP_3_ and TRP channels, which are responsible for the intracellular release of ER-resident Ca^2+^. In the 2-APB alone group, male mice exhibited non-significant increases in macrophage and PMN-MDSC populations, although trends were documented (**Figure 1B**). 2-APB resulted in similar levels of M-MDSC and neutrophils to mock samples. In female mice, 2-APB alone resulted in an increase in macrophages compared to the mock while neutrophil, M-MDSC, and PMN-MDSC numbers were lower compared to the mock (**Figure 1C**).

In our previous study, inhibition of Ca^2+^ signaling was reported to be associated with alterations in histological and inflammatory markers following vaping and infection exposure (31). In the presence of 2-APB, male and female mice demonstrated a significant decrease of neutrophilia compared to vape+LPS exposure (**Figures 1B, C**). A decrease in the PNM-MDSC population was also observed. However, this decrease was only significant in females. In all mice, the vape+LPS dual exposure led to a decrease in macrophage number, which in the presence of 2-APB was significantly reversed to higher levels similar to vape alone exposure.

### 3.2 Vaping and LPS challenge leads to multiple observed changes in cytokine burden

Because of the cellular activation and chemotaxis differences observed from our flow cytometry analysis with regards to immune cell populations, we also questioned whether differences could also be detected among the cytokine profiles. BALF samples were first collected and clarified. The clarified BALF supernatant was used for BCA total protein analysis and a MILLIPLEX panel of cytokines. PBS control values for cytokine burden were below quantifiable level. As such, these samples were removed from display within the cytokine panel. This exclusion is due to a technical limitation related to low protein yield from BALF rather than an absence of cytokine production.

**Figure 2:**
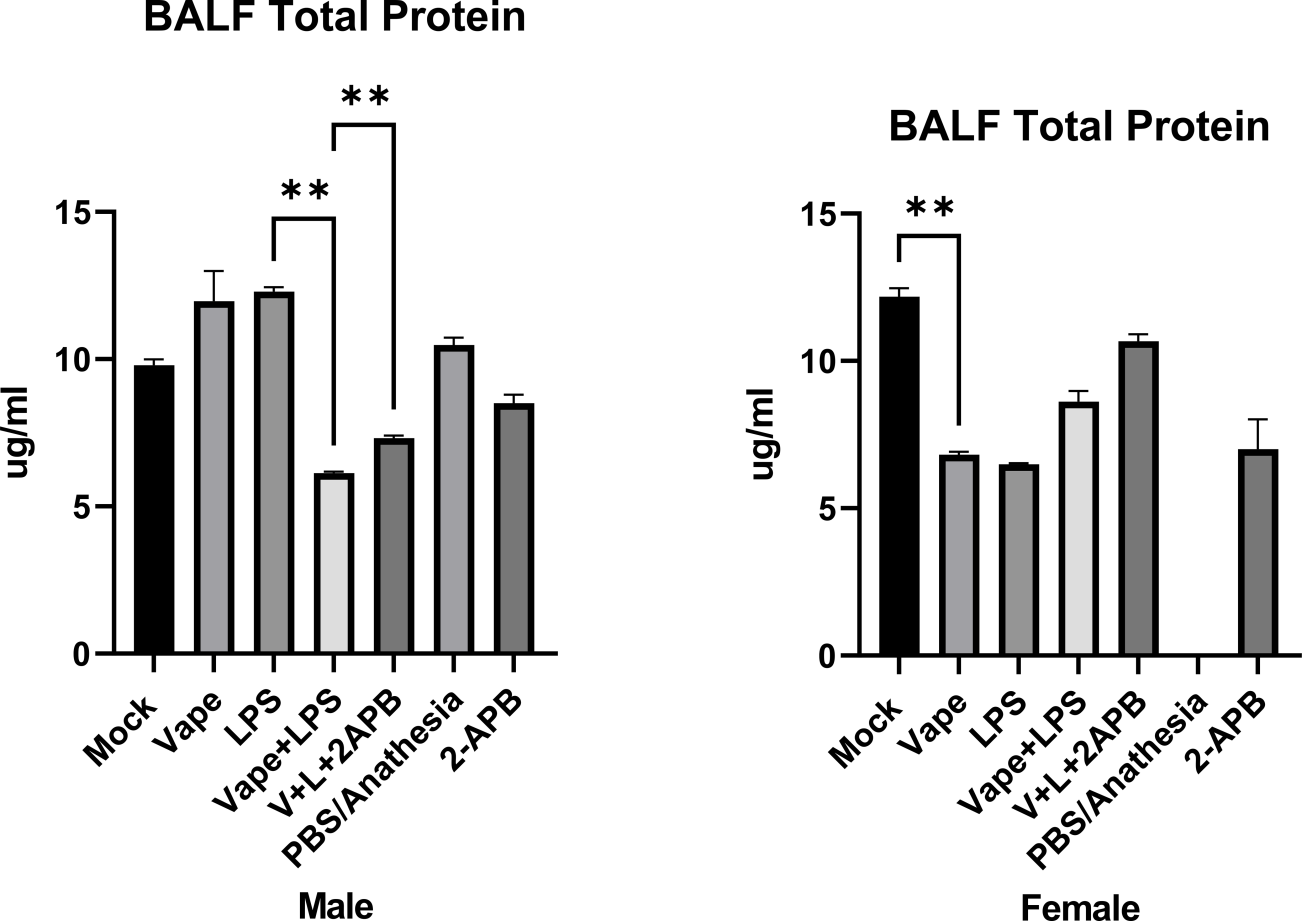
Total protein was analyzed from the clarified BALF of the experimental mice using Bicinchoninic acid (BCA) protein assay. BALF samples were collected from the male and female mice exposed to Mock, vape alone, vape+LPS and vape+LPS+2-APB conditions. PBS/Anesthesia group demonstrated protein readings below quantifiable level. This is most likely a technical issue arising from BALF extraction and isolation. Data are presented as mean ± SEM. Unpaired t-tests with Welch’s correction were used to determine statistical significance, which was indicated by the following p-values: P<0.01 **.

A bicinchoninic acid (BCA) protein assay demonstrated significant differences in total protein present in samples after treatment. BCA shows that in male mice, the vape alone and LPS groups demonstrate the highest level of total protein with a significant decrease noted in vape+LPS vs. LPS alone (**Figure 2**). Male mice exposed to vape+LPS exhibited lower total protein levels compared with LPS alone, alongside with differences in cytokine levels. Female mice exposed to vape+LPS exhibited differences in inflammatory markers compared with vape alone and LPS alone groups, although this initial protein analysis did not reach statistical significance. These data suggest that females exhibit higher inflammatory markers while males show lower levels of inflammatory markers after vape+LPS exposure.

**Figure 3:**
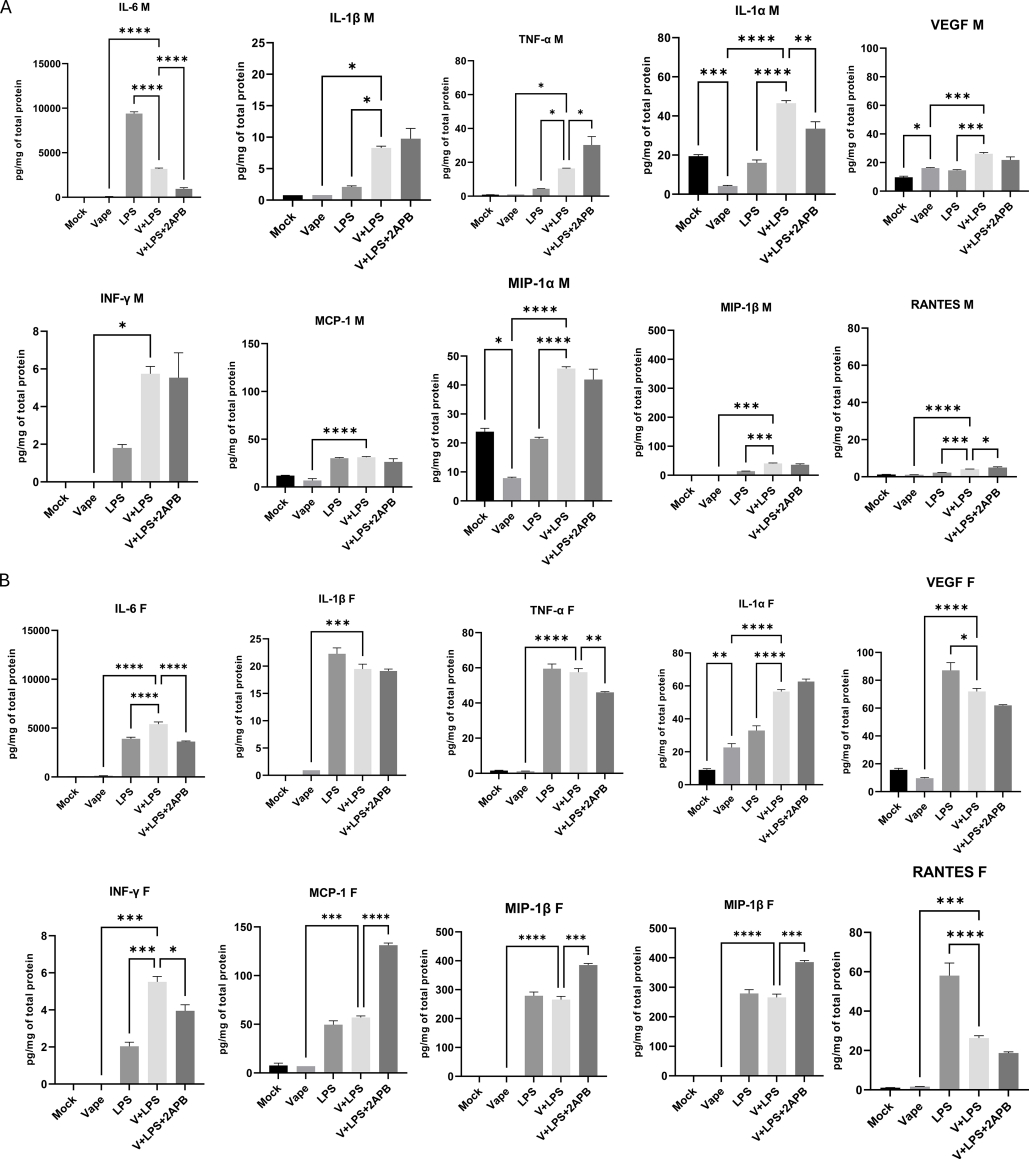
Cytokine concentration of clarified BALF from **(A)** male (M) and **(B)** female (F) mice was measured utilizing a 25 cytokine MILLIPLEX (MCYTOMAG-70K-PMX, Millipore-Sigma) panel and quantified using a Luminex LX200 system. BALF samples were collected from the male and female mice exposed to Mock, vape alone, vape+LPS, and vape+LPS+2-APB conditions. Inflammatory cytokines analyzed are characterized by classification in **Table 2**. Data are presented as mean ± SEM. Unpaired t-tests with Welch’s correction were used to determine statistical significance, which was indicated by the following p-values: P<0.05*, P<0.01 , P<0.001, P<0.0001***.

Similar to the differences in cellular activation and chemotaxis observed above (**Figures 1B, C**), cytokine burden did vary among mice that were vaped or received the LPS challenge vs. those that were vaped and received the LPS challenge (**Figures 3A, B**). As expected, pro-inflammatory cytokine (IL-1α, IL-1β, IL-6 and TNF- α) burdens were significantly higher after the LPS challenge. Each of these cytokine levels was significantly increased for the male mice receiving the dual vaping and LPS challenge exposure with the exception of IL-6 while IFN-γ, a pleiotropic mediator of (1) MDSC activation, i.e., immunosuppression, and (2) macrophage activation (40), was significantly upregulated by a dual vaping and LPS challenge exposure in both male and female mice (**Figures 3A, B**). Adding to this heterogeneous cell population, several chemoattractant cytokine levels (MIP-1α, MIP1-β and RANTES/CCL5) were also significantly increased for the male mice receiving the dual vaping and LPS challenge exposure vs. LPS only (**Figure 3A**). In female mice, the chemoattractant RANTES/CCL5 demonstrated a significant decrease in mice receiving the dual vaping and LPS challenge exposure vs. LPS only (**Figure 3B**). These data, again, indicate a disparity in terms of the overall response to the dual modality of vaping and LPS challenge vs. either vaping alone or LPS challenge alone.

### 3.3 Vaping and LPS challenge leads to an increase in acute lung injury

**Figure 4:**
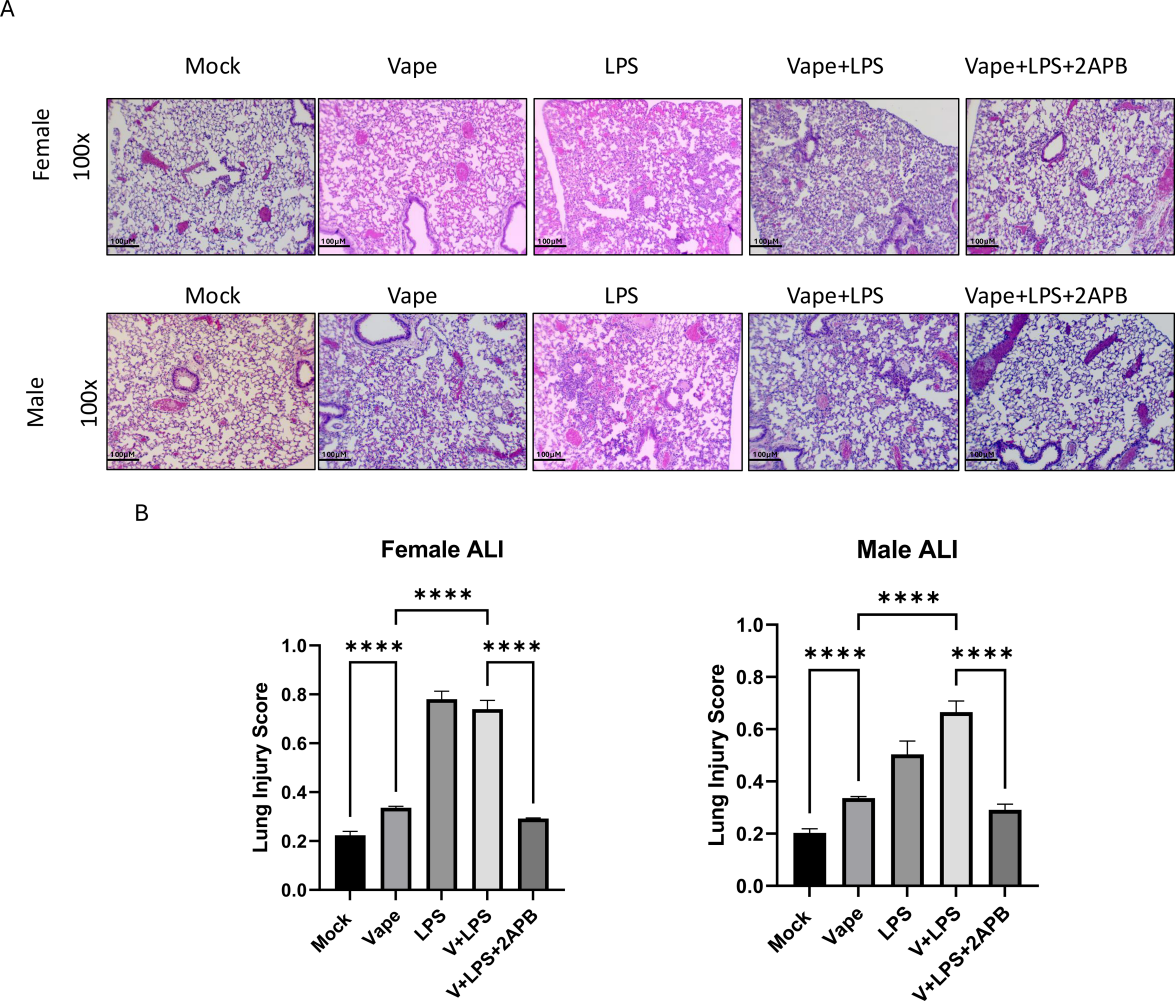
Lung histopathology analysis following vaping and LPS exposure of male and female mice. **(A)** Displayed H&E stained lung tissue from male and female mice were isolated from mock, vape alone, LPS alone, vape+LPS and vape+LPS +2-APB. **(B)** Acute lung injury (ALI) scores were generated and quantified by using 5 parameters as described in the materials and methods. Data are presented as mean ± SEM. Unpaired t-tests with Welch’s correction were used to determine statistical significance, which was indicated by the following p-values: p<0.00001****.

Lastly, based on differences seen in cellular populations as well as inflammation we wanted to investigate the direct influence on lung morphology. To this end, H&E staining of sections of whole lung tissue from mock, vape alone, LPS alone, vape+LPS and vape+LPS+2-APB were evaluated for injury by ATI scoring as previously described (39). Initial histological comparisons demonstrated that, in both male and female mice, LPS alone and vape+LPS were associated with more pronounced histological changes than mock and vape only, characterized by thickening of alveolar walls and neutrophilia (**Figures 4A, B**). In addition, 2-APB treatment was associated with differences observed in histological outcomes and inflammatory markers in both male and female mice.

## 4 Discussion

Recently, we (and others) have shown that vaping likely is a significant mediator of pro-inflammatory responses that may contribute to increased pathology and disease (2, 41–44). Likely and most dramatically, during the mid- to late- 2019 several outbreaks of an acute respiratory illness were demonstrated and then reported by those utilizing e-cigarette devices. These patients suffered from a mysterious severe respiratory distress that occurred acutely after utilizing e-cigarette products, with this phenomenon since termed electronic cigarette or vaping use-associated lung injury (EVALI) (3, 45, 46). EVALI outbreaks in the past have been linked to vitamin E acetate in products. However, these data may still be relevant in interpreting our JUUL menthol immune cell modulation paradigm because other compounds found in vapes could still be leading to lung injury. Overall, e-cigarette exposure has been shown to lead to the secretion of proinflammatory cytokines within the lung (2, 11, 17, 27). In particular, multiple immune cell types are affected and either activated or immunomodulated in some manner. Further, e-cigarette exposure has been demonstrated to increase the expression of pro-inflammatory cytokines such as IL-8 (11). An early study utilizing e-cigarette vapors and epithelial cells cultured at an air-liquid interface also resulted in the subsequent release of monocyte and neutrophil chemokines, demonstrating a direct effect for e-cigarette exposure in inducing inflammatory responses (47). However, *in vitro* e-cigarette aerosol exposure has also been documented to inhibit the phagocytic and efferocytic abilities of primary macrophages, leading to an observed decrease in clearance when a bacterial challenge model was presented (48). Similar results have also been obtained when exposing neutrophils to these aerosols, i.e., e-cigarette aerosols decreased the phagocytic and bactericidal activities of neutrophils (49, 50). As such, suppressing the antimicrobial functions of both macrophages and neutrophils by e-cigarette aerosols *in vitro* and *ex vivo* supports the concept that e-cigarette use damages host defenses and will likely result in an increased susceptibility to pulmonary infections.

Here, we have focused on MDSCs, which are a heterogeneous population of cells that are generated, and expand, during inflammation and infection alongside macrophages, neutrophils and dendritic cells, all of which are considered important immunoregulators (20, 21, 51). Our current findings demonstrate alterations in immune cell populations after mice are exposed to vaping and LPS compared to vape alone. The vape+LPS exposed male and female mice displayed a decrease in macrophage number as compared to the LPS exposure alone or vape alone. However, an increase in the M-MDSC population was observed with the male LPS only exposure (**Figures 1B, C**). Notably these M-MDSC populations show a decrease compared to the mock. PMN-MDSC numbers demonstrated a slight, but significant, decrease in number after the dual vape+LPS exposure compared to LPS alone. However, this trend was only observed in females. Males did not demonstrate a significant difference in PMN-MDSC populations after the vape+LPS exposure compared to LPS alone. LPS has previously been shown to induce the activation of toll-like receptor 4, a key mediator in the innate immune response (52). This activation leads to the release of inflammatory mediators such as granulocyte-macrophage colony-stimulating factor (GM-CSF) and interleukin-6 (IL-6), whose production activates signal transducer and activator of transcription 5 and 3 (STAT5 and STAT3), which in turn leads to MDSC generation (53–56). Notably, several inflammatory cytokines linked to MDSC generation were observed after LPS exposure only, specifically IL-1β, IL-6 and VEGF (**Figures 3A, B**). In the male mice, these cytokines demonstrated increases in IL-1β and VEGF in vape+LPS compared to LPS alone. In contrast, the levels of those specific cytokines were lower for the dual vape+LPS exposure group in female mice, which may suggest some blunting of the immune response. Though these data were significant, further analysis is needed to confirm these cytokines’ direct correlation with MDSC populations.

A similar blunting of the immune response has also been documented among COPD patients. Previous research has shown that COPD patients demonstrate increases in MDSC activation and, after 4 months of cigarette smoke exposure, an increase in MDSC numbers present in bone marrow, spleen and lung (18, 57). These MDSCs represent population differences that coincide with altered inflammatory markers, and these data suggest an association between and MDSC alterations and broader immune cell population changes. Previous research in the field of vaping has demonstrated that vaping results in a profound inflammatory response and various effects in the cell. Chitteti et al. demonstrated that exposing cells to the plasma of e-cigarette users results in various levels of cellular stress (58). They found that metabolic stress was increased and the overall function of the mitochondria in epithelial and endothelial cells was changed. Specifically, vaping led to structural changes in the mitochondria, resulting in increases in mitochondrial membrane potential (MMP) and reactive oxygen species (ROS). This result is similar to the effects of tobacco smoke, suggesting that mitochondrial function may also play a role in the vaping-dependent disease paradigm. Additionally, ROS have been implicated in the regulation of Ca^2+^ signaling pathways; however, the relevance of this pathway to MDSC population changes in the context of vaping exposure was not directly explored in this study and remains speculative. Tobacco/cigarette smoke is known to inhibit the host’s immune response. However, the effects of e-cigarettes on the immune response are relatively understudied. Martin et al. demonstrated in epithelial cells in the nasal passage that vaping led to the suppression of a variety of immune-related genes that are also suppressed in cigarette smokers (12). This study also found that e-cigarette use not only lead to a greater level of suppression in genes compared to smoking but also a larger variety of genes suppressed. Early growth response 1 (EGR1), which is a mediator of various cellular responses, was shown to have a 10-fold decrease in expression in e-cigarette users compared to tobacco smokers. Additionally when looking at functional genes associated with reduced EGR1 expression, e-cigarette users displayed 18 genes compared to the 5 found in cigarette smokers. These data are consistent with immune-related changes observed in various tissues exposed to e-cigarettes.

In conclusion, our model builds upon our previous *in vivo* research demonstrating that vaping exacerbates lung cellularity and inflammation in the presence of an infection (i.e., LPS) (31). The exact role vaping plays in immune response and regulation is relatively unknown. However, these data suggest that in an addition to modulating Ca^2+^ signaling (30, 31) that vaping may have some effect on MDSC generation, activation and migration. To our knowledge our paper represents a novel investigation of MDSCs in vaping and pulmonary diseases.

Our data demonstrate decreases in macrophage and M-MDSC numbers in male mice dually vaped and challenged with LPS compared to LPS alone. Vape+LPS exhibited slightly higher levels of M-MDSCs compared to vape though this comparison is not significant. In female mice, neutrophils, macrophages and PMN-MDSC demonstrated decreased populations after vape+LPS exposure compared to LPS alone. These effects appear to be diminished or reversed in the presence of 2-APB. Paired with our cytokine and chemokine level data, these results suggest that vaping exposure is associated with alterations in M-MDSC, macrophage and neutrophil populations as well as differences in inflammatory mediator levels. Given that this study explored multiple experimental variables, pairwise statistical comparisons were used to examine the differences between experimental conditions, and future studies will be needed to assess direct interaction effects among treatment variables. PMN-MDSCs and neutrophils share overlapping phenotypes, and their representation should be considered when interpreting differences in inflammatory marker profiles (59). Potentially this dysregulation could be pharmacologically corrected using Ca^2+^ signaling inhibitors though this is a major limitation of our study. 2-APB is a non-specific inhibitor with characterized off-target effects. In the absence of direct measurements of intracellular Ca^2+^, conclusions related to Ca^2+^ signaling remain exploratory. Further investigations of the mechanisms of Ca^2+^ signaling inhibition are needed to validate the pharmacological relevance of this study. That said, it appears MDSCs may play a role in Ca^2+^-mediated pulmonary immune cell modulation. As such, these data then further underlie a need for a better overall understanding of the mechanisms leading to vaping-dependent pulmonary disease and inflammation.

## Data Availability

The raw data supporting the conclusions of this article will be made available by the authors, without undue reservation.

## Supplementary material

Supplementary Figure 1 Flow cytometry analysis of BALF to identify immune cell population in the lungs. Cells were stained with fluorescent antibodies to identify macrophages, neutrophils, M-MDSCs and PMN-MDSCs. Gating strategy SSC-A vs FSC-A plot: for gating out clumps and debris. SSC-A vs PB450-A plot: To gate PB450-A+ cells out of following analysis. PE-A vs APC-A750-A plot: For distinguishing macrophages and neutrophils. Neutrophils separated based on LY6G+/F4/80- characteristics while macrophages were distinguished by F4/80+/LY6G- characteristics. SSC-A vs APC-A plot. CD11b+ was used to initially distinguish MDSC populations. FITC-A vs PE-A plot: CD11b+ cells from previous plot were considered MDSC cell population. Ly6G and Ly6C were used to further separate MDSCs into M-MDSC (Ly6G-/Ly6C low) and PMN-MDSC (Ly6G+/Ly6Clo).
