# Supplementary figures and images for: Vaping induces a marked pro-inflammatory state within the lung, which is at least partially due to its immunomodulatory effects
Source: Front Immunol. 2026 Mar 10;17:1759361. doi: 10.3389/fimmu.2026.1759361 (PMC13008690; doi:10.3389/fimmu.2026.1759361)

A

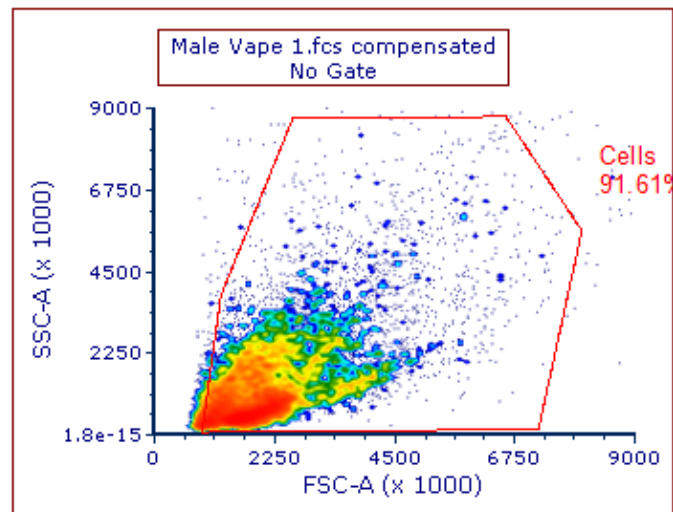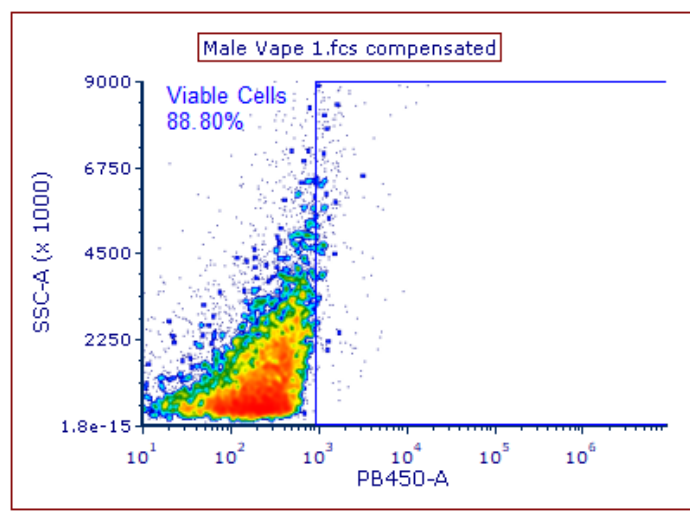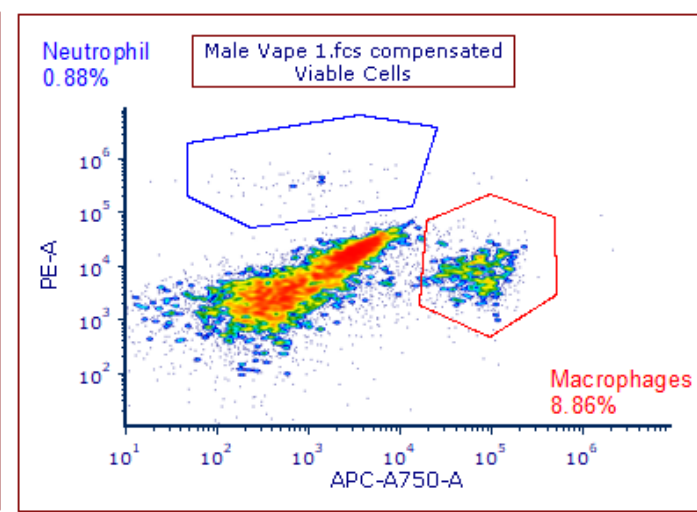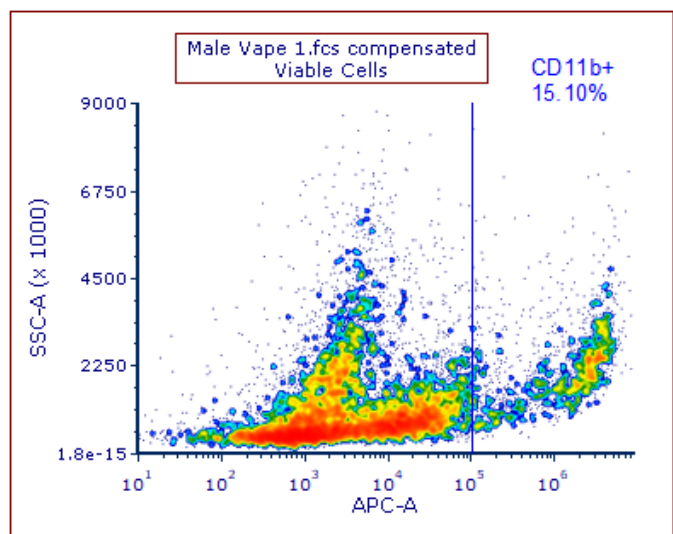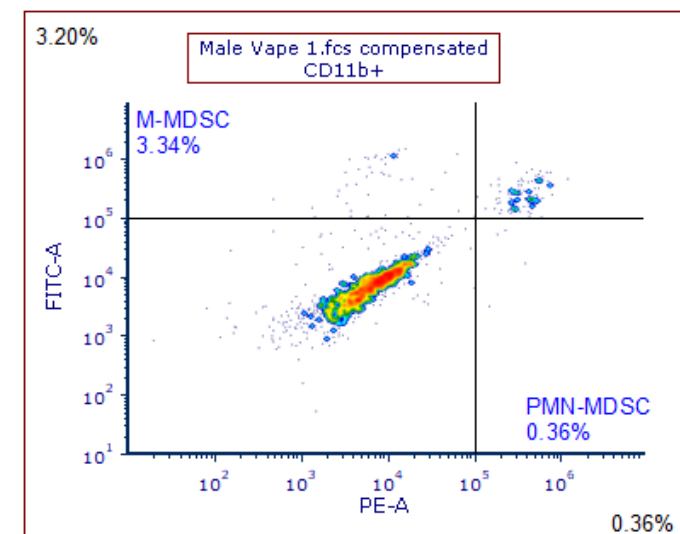

Supplement: Supplementary Figure 1 — Flow cytometry analysis of BALF to identify immune cell population in the lungs. Cells were stained with fluorescent antibodies to identify macrophages, neutrophils, M-MDSCs and PMN-MDSCs. Gating strategy SSC-A vs FSC-A plot: for gating out clumps and debris. SSC-A vs PB450-A plot: To gate PB450-A+ cells out of following analysis. PE-A vs APC-A750-A plot: For distinguishing macrophages and neutrophils. Neutrophils separated based on LY6G+/F4/80- characteristics while macrophages were distinguished by F4/80+/LY6G- characteristics. SSC-A vs APC-A plot. CD11b+ was used to initially distinguish MDSC populations. FITC-A vs PE-A plot: CD11b+ cells from previous plot were considered MDSC cell population. Ly6G and Ly6C were used to further separate MDSCs into M-MDSC (Ly6G-/Ly6C low) and PMN-MDSC (Ly6G+/Ly6Clo). [file Image1.pdf]

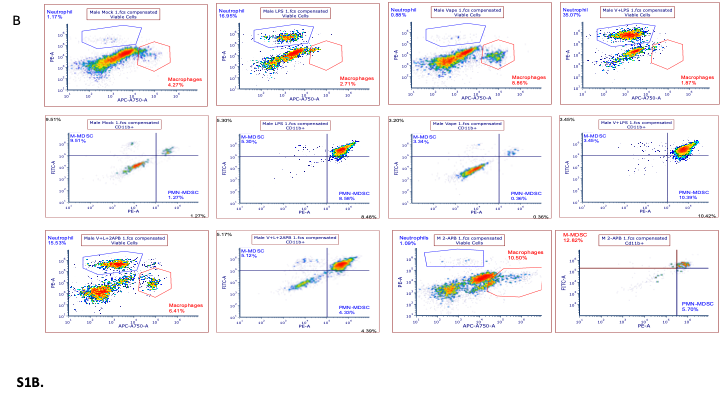

Supplement: Supplementary file 2 [file Image2.tiff]

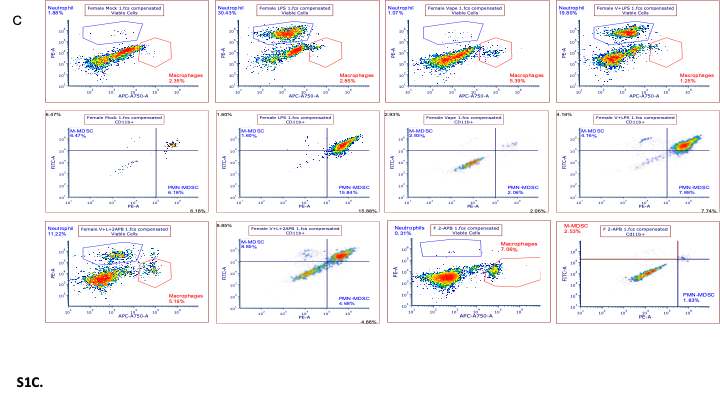

Supplement: Supplementary file 3 [file Image3.tiff]
